# Exploring the role of community-based knowledge brokers in public health communication through the lens of self-determination theory: a qualitative study

**DOI:** 10.1186/s12889-026-28665-7

**Published:** 2026-08-06

**Authors:** Oliver Clabburn, Fiona Gillison, Imogen Coningsby, Paul Scott, Ottilie Kuehne, Julie Barnett

**Affiliations:** 1 Bath, England, UK; 2 Department for Health, Centre for Motivation and Behaviour Change, University of Bath, Bath, England, UK; 3 Public Health, Bath & North East Somerset Council, Bath, England, UK; 4 Department of Psychology, University of Bath, Bath, England, UK

**Keywords:** Community, Broker(s), Public Health, Self-Determination Theory, Communication, Information, COVID-19, Local authority

## Abstract

**Background:**

Encouraging people to change their behaviour through public health messaging is challenging, and during the COVID-19 pandemic, many communities reported barriers adhering to government regulations and guidance due to the accessibility, understanding or mistrust of information. Stakeholders within community organisations have been recognised as potential brokers between Public Health authorities and communities. This study took a theory-informed approach to explore the ways in which brokering may work, through the example of the communication of health protection messaging during the COVID-19 pandemic.

**Methods:**

We adopted a qualitative cross-sectional study design using semi-structured interviews with key informants from the voluntary, community, faith and social enterprise sector (VCFSE), local private sector, schools and parish councils in the South West of England. Interviews were conducted online, and interview transcripts analysed using inductive thematic analysis. Themes were then interpreted through the lens of Self-Determination Theory (SDT), identifying factors consistent with providing support for basic psychological needs (autonomy, competence and relatedness).

**Results:**

Three themes were developed to describe community organisation actions in communicating public health messages to their communities; i) the emergence of a brokering function as a response to mistrust and misinformation; ii) tailoring information according to hyper-local community needs and differences, and iii) a perceived need for power to be delegated to the local level.

**Conclusions:**

Community-brokers play a vital role in public health communication during crises. Applying a lens of self-determination theory provided a framework for organising the aims and techniques used by stakeholders to promote behavioural adherence within their communities. The descriptions stakeholders gave of their actions aligned with recommended techniques for supporting autonomy (e.g. providing choice, a tailored rationale, and recognising barriers), competence (e.g. tailoring instructions, providing support) and relatedness (e.g. emphasis of shared values and benefits). Policy frameworks and preparedness for future public health emergencies should acknowledge the role of community-brokers and include appropriate mechanisms to enable enhanced support of their communities.

**Supplementary Information:**

The online version contains supplementary material available at https://doi.org/10.1186/s12889-026-28665-7.

## Background

Throughout the COVID-19 pandemic, communities across the globe experienced unprecedented changes to everyday life. As knowledge about COVID-19 increased, it was apparent that societal behaviour change would be fundamental to mitigate the spread of the disease. Governmental guidance was informed by emerging research evidence which paved the way for numerous behaviour adaptations such as wearing face coverings, handwashing, social distancing and isolation. The messaging disseminated was: “Stay at home. Protect the NHS. Save lives” which aimed to protect society through enforcing binary and universal rules, applicable to all people throughout the nation [1]. In reality, however, not everyone adhered to The Health Protection (Coronavirus) Regulations 2020 [2]. The aim of this paper is to explore the role of community-based stakeholders who acted as intermediaries, brokering the transfer of information from national public health policy to local communities.

In keeping with emerging UK health policy, we use the term ‘hyper-local’ in reference to the immediate neighbourhoods and communities in which people live and interact. Typically smaller than statutory service footprints, they are shaped more by social relationships and everyday interactions than administrative boundaries. This concept aligns with the increasing emphasis on neighbourhood working within the NHS 10 Year Plan [3] and emerging integrated neighbourhood models [4].

### Brokers

We are using the concept of community brokers to refer to individuals or organisations who mediate health and social care advice to local communities or individuals. One such example is community-health workers (CHW) who strive to improve the health of their local community, particularly in low- and middle-income countries [5]. According to Schaaf and colleagues [6], CHWs bridge between formal public health officials and community members via three functions: (i) service extender: bringing health services to community members to support uptake, (ii) cultural broker: communicating health priorities and information through culturally responsive and accessible methods, and; (iii) social change agent: championing and advocating on behalf of their local community [6].

Prior to the COVID-19 pandemic, community champions or health ambassadors were established as a means to tackle health inequalities at the grassroots level across England [7]. Drawing on life skills, local knowledge, established relationships and embedded trust, the focus was on place-based health promotion with an aim to “improve the health and wellbeing of their families, communities or workplaces” through bridging between health services and communities (p.36) [8]. Alongside *health promotion* is the role of *health communication* which is key to community-centred approaches to public health [9]. Related literature suggests that stakeholder actions are effective due to embedded knowledge about their local community, understanding the issues and priorities of local people and using appropriate communication methods [10].

The onset of COVID-19 brought the concept of community brokers to the forefront of emergency response to the pandemic in England. Traditional top-down approaches for public health communication were impeded due to an ‘infodemic’, fuelled by misinformation, complex messaging and wide-spread mistrust [11]. Subsequently, programmes such as COVID Champions were developed whereby information was brokered and disseminated to communities through local trusted stakeholders [7]. This approach acknowledged the power of local knowledge within champions, knowing how to best help, support and engage their respective communities [12].

For many, knowledge was brokered through community leaders. Purewal and colleagues investigated the role of civil society organisations (also known as not-for-profits, or VCFSEs in England) in disseminating COVID-19 information to community members in British Columbia (Canada) whose first language was not English [13]. Community-based knowledge brokers played a crucial role in health communication by facilitating multilingual and culturally-appropriate information to reach community members. Although the study was situated in Canada, the authors suggest relevance and transferability to other settings, recognising the important role of community-based knowledge brokers in public health communications [13].

### Self-determination theory

Applying theory to explore the mechanisms through which positive outcomes are brought about can help to create transferable knowledge, and may help to identify lessons learnt from the COVID-19 pandemic that can influence future practice. Self-determination theory (SDT) is a meta-theory of human motivation and functioning which is centred around the quality, rather than quantity, of motivation. The quality of motivation is considered to lie along a continuum from the least to the most self-determined. At one extreme, behaviour feels like it is controlled by others (external regulation, acting to avoid punishment or gain reward), and the other extreme, behaviour is described as intrinsic, where action is taken purely for enjoyment or its own inherent rewards. Three types of regulations lie between the two motivation extremes: Introjected (acting to avoid guilt or shame, or to gain approval), identified (acting to achieve personally meaningful, but still extrinsic consequences), and integrated (having assimilated the reason to act with one’s values and identity) [14]. These different types of regulation can be grouped into two categories of controlled (external and introjected) and autonomous motivation (intrinsic, integrated and identified). Such distinctions are important; autonomous forms of motivation typically result in sustained, robust behaviour, which has a positive impact on affect and wellbeing, whereas actions carried out as a result of controlled motivation tend to be shorter lived, poor quality (undertaken half-heartedly, cutting corners) and associated with negative emotions such as frustration and negative affect.

Promoting behaviour change through autonomous motivation can take time, such that when an immediate response is necessary, as was the case during the COVID-19 pandemic, mandatory rules may need to be enforced using more controlling practices (e.g. penalties). Given individuals do not have agency over whether to adhere, this approach would be expected to lead to controlled motivation. If regulation remains controlled, it is likely that adherence would be short lived, however, rules can be internalised to become more autonomous over time if accompanied by a meaningful rationale and respect for the challenges people may experience in adopting them. That is, we may feel autonomous and volitional in adhering to rules if we believe they are necessary, proportionate, and justified for our benefit. SDT sets out the mechanism through which behaviour can be internalised in this way as the process of satisfying three universal basic psychological needs; the need for *autonomy*, feeling that one has choice and is the origin of one’s actions, *competence*, feeling that one has the capacity and opportunity to act in a given way, and *relatedness*, feeling that acting in a given way brings you closer to valued others and enhances a sense of belonging [14]. Through exploring how people’s psychological needs (in a given environment) are supported or frustrated, SDT can be a useful theory to use in mapping attitudes, barriers and facilitators of compliance in order to design strategies for promoting adherence in public health.

A large-scale longitudinal study in Belgium provided support for the application of SDT at this population level, finding autonomous motivation to be significantly associated with COVID-19 adherence in both the short and long term, while external regulation did not predict long term outcomes and was negatively associated with adherence in the short term [15]. However, most research exploring mechanisms and techniques for promoting autonomy support relates to individual behaviours, or organisational level (e.g. school [16] and workplace [17] policy) with little research conducted at the national policy level. Nonetheless, during COVID-19, recommendations to public health teams were published demonstrating how the principles of SDT could be applied to public health messaging in order to promote motivation for adherence that was more autonomous – and less controlled, mirroring the techniques show to support internalisation (i.e. shift from more controlled motivation to more autonomous in adhering to personal health advice) at the individual level [11, 18, 19]. For example, Porat and colleagues [11] conducted an evidence synthesis resulting in five guidelines to cut through what they termed as an ‘infodemic’ (excessive and sometimes misleading information) at the community-level: (1) create an autonomy-supportive health care climate; (2) provide choice within limitations; (3) apply bottom-up (vs. top-down) communication using principles of co-production; (4) create solidarity (we are all in this together), and; (5) be transparent and acknowledge uncertainty [15]. Further elaboration of how people’s autonomy can be supported in the promotion of COVID-19 regulations was provided by Martela and colleagues [19], who refer to providing “autonomy supportive limit setting” (ensuring a clear rationale is provided for the specific limits set) along with assurance that laws will be enforced fairly. They acknowledge that the success of this approach may depend on the degree to which people believe rules are coming from a legitimate and trustworthy source and see their leaders modelling recommended guidelines, though there has been limited research on this conducted at the policy level [19]. While non-adherence from some government leaders was highly visible in the UK during the COVID-19 pandemic [20], community brokers were often perceived as trusted local leaders whose behaviour was less publicly scrutinised and whose legitimacy was grounded in established relationships within their community.

Our study aimed to provide this insight by applying the lens of SDT to the experiences of community organisation brokers in the South West of England. We examined the ways in which public health communications were brokered through community organisations, to better understand how interactions supported, or potentially thwarted, the psychological needs for autonomy, competence and relatedness. Our intention was to provide insight on the mechanisms that may have shaped how community brokers supported public engagement and behaviour change.

## Methods

### Study design

Qualitative analysis of interview data originally collected during the COVID-19 pandemic. The original study comprised semi-structured interviews with key informants from organisations that maintained regular contact with local communities, and the present analysis sought to explore these data through the theoretical lens of Self-Determination Theory.

### Setting

Situated within the context of the COVID-19 pandemic, a local authority in the South West of England investigated the efficacy of public health communication, guidance and messaging. Specific focus was directed towards how people might be further enabled and encouraged to follow national guidance on social distancing, testing, and self-isolation. Data were collected between November and December 2020 while the UK was subject to the second national lockdown. At this time, the local authority shared COVID-19 guidance and information with stakeholders through a variety of methods including third sector newsletters, networks and webinars.

### Sampling and recruitment

A convenience sampling approach was adopted. Potential participants were identified by the local authority through existing networks and mailing lists of organisations actively engaged with local communities during the pandemic. Eligible organisations included VCFSE groups, schools, parish councils, local businesses, housing and other community-facing services. Invitations were sent to over 800 email addresses across the local authority area. Participants were identified due to their longstanding relationships and regular engagement with their communities, as opposed to any formal public health or knowledge-broker role.

### Procedure

Stakeholders were invited to take part via email from a member of local authority staff, which included study information and participant sheet. Volunteers were assured that their contributions would remain anonymous and were asked to provide informed consent by email prior to the interview. According to participant preference, video-interviews were conducted via Zoom or Microsoft Teams by two researchers (IC/OK) that had undergone research training at the University and were supervised by an experienced researcher (JB). A verbal reminder of the purpose of the study was given before the interview commenced, as well as an opportunity to ask further questions, and a reminder of their right to withdraw participation without needing to give a reason. With permission, all interviews were audio-recorded and professionally transcribed. No financial incentives or reimbursement were provided for participation. Participants were informed that the findings would contribute to the design of future local public health communications and interventions.

Interviewers followed a topic guide (Additional file 1) which was developed by the research team with reference to the study aims and existing published literature (PS/JB/IC/OK). The topic guide was intentionally broad to allow participants to bring in any aspect of their own and others’ experiences as they felt were relevant. For each of three key behaviours - social distancing, self-isolation and testing - participants were asked to report on how the communities they worked with (i) coped the regulations, (ii) how well they followed the guidance and why, (iii) whether adherence changed over time, and what (iv) helped and (v) hindered adherence.

### Ethics

The study was approved by the University of Bath Psychology ethics committee (ref:0924 − 807) and adhered to the principles within the Declaration of Helsinki.

### Data analysis

Interview transcripts were anonymised before reflexive thematic analysis was conducted, informed by the six-phase approach [21, 22]. One researcher (OC), who had not been involved in data collection undertook an inductive analysis of the complete dataset [21]. Familiarisation was achieved through repeated, line-by-line reading of each transcript. Reflections and conceptual interpretations were recorded in a reflexive journal. Initial codes were generated inductively from the data which were iteratively reviewed and organised into preliminary themes. Repeated engagement with the dataset and discussions with the wider research team (PS/JB/IC/FG) ensured coherence, internal consistency and ongoing relevance to the research question.

Taguette [23] software was used to manage data whilst also retaining the richness and complexity of each participant’s contribution [24]. Rather than using the concept of saturation, the themes we identified reflect the quality of the information that participants provided, the recurrence of relevant data, and the extent to which these data offered insights that addressed our research question [25, 26].

Following development of the final themes, findings were interpreted through the lens of SDT. This theoretical interpretation was undertaken collaboratively between OC and FG, whose expertise in SDT informed consideration of how the themes related to the psychological needs for autonomy, competence and relatedness. SDT was therefore used to interpret the inductively derived themes rather than to guide data coding.

## Findings

Interviews were carried out with 23 stakeholders who represented 22 organisations from the voluntary, community, faith and social enterprise sector (VCFSE) (*n* = 10), parish councils (*n* = 4), local authority (*n* = 3), private sector (*n* = 3) and schools (*n* = 2). All took place in November and December 2020 with duration ranging from 23 to 54 min (mean = 39 min). Three overarching themes were developed: (i) Emergence of a brokering function as a response to mistrust and misinformation; (ii) Tailoring information according to hyper-local community needs and differences, and (iii) A perceived need for power to be delegated to the local level. Each will be discussed in turn with use of anonymised participant extracts to illustrate each theme.

### Theme 1: Emergence of a brokering function as a response to mistrust and misinformation

Stakeholders working in front-line organisations felt that messaging about Governmental guidelines was perceived to be inconsistent, confusing and contradictory, which impeded their own, and their clients’ accommodation of rules:

“It’s too bloody complicated, I find. You can do this today, but you can’t do it tomorrow, or you couldn’t do it yesterday. At one point it’s like, “Right it’s too complicated we’re going to make it easier,” and then the next thing is, “Right we’re going to tighten up on the tiers,” whatever that’s going to look like. “What is a household and a bubble?” “Can we do this, and can’t we do that?” (O11: Drug & Alcohol Services)

Added to this inconsistency were perceptions that Public Health authorities and key authoritative figures within the four nations of the UK were interpreting and flexing national guidance. A rhetoric of ‘open for your own interpretation’ cascaded throughout communities, fuelled by inconsistent and poorly communicated messaging:

“I’d say quite a lot of the government messaging has been very confusing and I think that has given people a reason or a justification for interpreting them in a way that they think is sensible, and also I think the whole Dominic Cummings^1^ thing really undermined the view of rules being absolute.” (O9: Parish Counsellor)

Stakeholders felt that the national guidance had become less effective over time due to messaging which they perceived to be repetitive, complex and irrelevant. People leading local organisations observed that this resulted in a sense of cognitive fatigue, which in turn led to people seeking guidance from alternative sources they deemed to be trustworthy.

“Other families that we’ve worked with who are quite vulnerable, who maybe distrust messages that come from positions of authority, be it government, local council, whatever, if you’re telling them they can’t do something, their immediate reaction is to question that of “If I’m being told to, I’m going to do exactly the opposite”. So completely different ends of the spectrum, well both experiences of social distancing but for very different reasons.” (O10: VCFSE_Children & Families)

They described how a ‘community-broker’ role emerged whereby hyper-local representatives from VCFSE organisations, parish councils, schools and faith settings became preferred conduits of information between government and communities:

“We are part of the outlet of information as well because we will see a direct spike in calls and contacts on our website to the [org] the day after there’s been an announcement.” (O16: VCFSE_Advocacy)

Stakeholders attributed the emergence of this role to the long-standing relationships they had within their respective communities. This firmly embedded reciprocal trust, alongside perceptions of community-brokers being separate from the government, meant they were treated as credible, empathetic and reflective of local community values:

“So, it [information] came from the parish communications’ email, which is the recognised official email address so it had immediate trust.” (O8: Parish Counsellor)

Added to this was an appreciation that community-brokers were ‘on the ground’ in the local area which promoted a sense of accessibility:

“There are around 2,000 community connectors that are connected through [org] who are part of the hub as well with us. They’re part of that organisation and they really are our frontline staff effectively, volunteers out on the ground. So, in some cases they could actually take or collect paperwork and leaflets to people or information that they needed to get distributed so they didn’t need to come into the one-stop shops and they didn’t need to have that face-to-face. So, that’s been one way that it’s been impacted and mitigated.” (O16: VCFSE_Advocacy)

Guidance originating from community-brokers was therefore perceived to be of importance and directly relevant to their community members. Stakeholders believed this increased the likelihood of accommodating guidance, adhering to rules, and ultimately - modifying behaviour:

“My role is to read messaging from the government and disseminate it [to…] the families we work with, I don’t think they’ve accessed the information effectively when there were daily news briefings. I know a lot of families tuned in and watched that, and that was quite useful while there was new information being handed out, although it became very, there was too much detail and a lot of families didn’t have a clue what was important and what wasn’t, so switched off.” (O10: VCFSE_Children & Families)

### Theme 2: Tailoring information according to hyper-local community needs and differences

The approach adopted by the community brokers recognised local diversity and nuance in explaining the interpretation of rules and guidance to their specific communities. Stakeholders felt that there was appreciation that community-brokers were ‘one of us’, in being from, and part of, the local area, subsequently possessing granular intelligence about their immediate locality. This may include, for example, understanding of differences in cultural attitudes and perceptions between adjacent local postcodes. With this degree of trust and legitimacy, brokers felt able to tailor the content and method of information dissemination in alignment with neighbourhood-based values, and saw themselves as curating information and support according to local preferences and needs:

“There’s always been a kind of feeling that the problem is caused by another group in society so it’s not your demographic that’s the problem, it’s another demographic. In [county], there was a feeling that it was students that were the cause of the problem which is not true and they have been pretty disciplined, I think. But that’s always the case, that people always see problems as being caused by other people and those other people could be another demographic or people who don’t live in [town], the problem is people, I don’t know, living in [neighbouring town] or [neighbouring city] or whatever.” (O4: Parish Counsellor)

National or Governmental advice was sourced by community-brokers, interpreted, and then refined into relevant and accessible formats for their respective communities. Community brokers would often draw upon underlying assets, knowledge and skills:

“What we’ve done is we’ve obviously got the information from the government and from [local authority] Public Health, which I am summarising and posting to the village. So, we have an internet group where everybody gets emailed and I summarise, because my background’s medical, I summarise it all for people and people can ring me if they’re worried.” (O2: Parish Counsellor)

Stakeholders commonly described information barriers for older people, teenagers, people living with disability or illness, and those with limited digital literacy. While online resources and Governmental publications were signposted in national communications as ‘go-to’ points of reference as the situation evolved, stakeholders considered that this was not appropriate for all community members due to limited access to technological devices, internet connectivity, digital literacy and reading comprehension of lengthy, complex publications:

“A lot [of our community members] wouldn’t have smartphones and even some who do, there’s quite a high level of illiteracy among my members. So anything like that just frightens them a bit, so rather than say, “I don’t have to do this” they will just go, “Oh no I don’t need that, I don’t do that, I don’t believe in it,” or something.” (O12: VCFSE_Food)

Stakeholders developed a role in providing targeted outreach, using tailored engagement methods:

“It’s just how that information is put out there, something that engages with the age groups that you’re aiming it towards, especially those younger people in terms of social media and how that gets to them. Someone my age would be watching the news and finding out what’s happening but I’ve got three kids myself. My 13 and 14 year old ain’t going to sit down and watch the telly. So it’s - can you provide some information on social media that they’re engaging with through schools, and what have you. That they’re making sure that the right messages are going out there. I think that’s the biggest thing, just how it’s ‘put out’ to people and that they’re aware.” (I5: VCFSE_Sport)

“We do have a parish wide email and that hits maybe 60% of the community, which is not ideal but it’s not bad. There are Facebook pages, WhatsApp pages, and emails to the zone coordinators, who print them off, so the parish emails are then delivered by hand to those who are not online. So everybody receives the communications the same.” (O8: Parish Counsellor)

### Theme 3: A perceived need for power to be delegated to the local level

Stakeholders described enforcing regulations within their organisation and frequently needing to develop their own. Many talked about the need to harness community pressure, as a more effective tool for individual behaviour change than national law:

“We have laws about dog fouling, we have laws about small society things but the laws are rarely enforced because the police aren’t there to enforce things. Somehow you have to communicate to people that they have to ‘buy’ into this idea, through a feeling that it’s the right thing for society […] sometimes laws have to be enforced by community pressure rather than police enforcement.” (O4: Parish Counsellor)

Further, they felt that delegating responsibility for enforcement to respective local authorities would support the development of more effective responses aligned with their nuanced understanding of community culture. For example, policing non-adherence via hyperlocal initiatives which focus on neighbourhood-based social pressure:

“They could have delegated to Parish Councils, they could watch over their patch […] It’s not monetary punishment but people being chastised within their community would have more impact than some remote £10,000 fine thing being put on them.” (O13: Parish Counsellor)

Stakeholders believed that individuals and communities would be more responsive if national guidance was framed through a lens of community cohesion and shared stoicism. They talked about how following the rules brought significant frustration to individuals’ lives, but they believed a local focus on communities’ sharing the experience of being ‘in it together’ was conducive to adherence:

“I think awareness has increased as time’s gone, shared responsibility has increased, understanding obviously is increasing day-by-day and so I think because of that, you’re just seeing a general groundswell, a general coming together of what we all need to be doing just to get through this and obviously the sooner we can do all these things, the sooner we can get through the worst of it and hopefully start to plan for some sort of new normal.”

(O1: Letting agent/property management)

Conversely, several stakeholders inferred that public fatigue and non-adherence were partly linked to a disconnect between national guidance and local experiences. Many community members felt their sacrifices were no longer recognised or making a visible difference to public health. Subsequently, national rules felt increasingly rigid, disempowering - and futile. Participants therefore described consoling members and having to adapt or modify messaging to ensure localised relevance:

“People have now got to a point where they don’t see the benefits of what their actions are doing, they’re not hearing about “you’ve stayed in for this amount of time, you’ve saved this many lives or you’ve saved your family”, it’s now “I want this and I’m going to do that because the guidance is changing and I don’t know why I have to follow it.” O10: VCFSE_Children & families)

Devolving power to community or neighbourhood level allowed local stakeholders to practically enact and apply their local knowledge and intelligence about the area. This lent itself to a holistic public health approach through a compassionate-community lens. Stakeholders were able to provide examples of how they had used data available to them locally to target support:

“We built up a list of who’s at risk, our definition of risk, not just the government’s, who needed what, because we wanted to make sure that everybody got what they needed to get through the initial lockdown. That included things like taking the bins out, would you believe it, gardening, shopping, and medication, that was the key thing.” (O8: Parish Counsellor)

With this in mind, suggestions were made that national Public Health guidance should not be perceived as concrete laws which apply in the same way to everyone, but instead should enable an approach which can be moulded according to individual needs and circumstances. They felt able to do that at the local level:

“I think a lot of personal circumstances actually make people do something slightly different. […] I think people have to have something really in terms of support from other humans, and that isn’t necessarily being able to, you know, not everybody has gardens and can go walking with somebody else or meet them during the day. People’s circumstances are incredibly uneven and unequal, and I’m sure that’s why in terms of more deprived areas and people who have less money, and all sorts of other difficult circumstances, find it almost impossible sometimes to follow, because it’s very, very difficult for some people.” (O9: Parish Counsellor)

## Discussion

Our findings reinforce international literature from Australia, America and Canada which highlight the important role of community-based stakeholders in disseminating public health information during the COVID-19 pandemic to their respective community members [13, 27, 28]. In the geographical area where the present study was conducted, community brokers were a conduit between national government policies and local communities. The first theme illustrates how community organisations emerged as brokers in response to national information that community members perceived to be inaccessible, unclear or untrustworthy, prompting increasing reliance on trusted local organisations as credible sources of information and support. The second theme illustrates how community brokers adapted guidance and advocated for adherence within their respective communities, and the final theme extends this to brokers’ perspectives that greater devolution of power over the implementation of public health guidelines to local areas could result in guidance that is more acceptable to communities, fostering greater adherence. Such findings reinforce existing literature emphasising the importance of local and trusted community sources in brokering information [13, 27].

Self Determination Theory (SDT) provides a useful framework to interpret our findings, particularly through considering the concepts of support for autonomy, competence and relatedness in relation to the actions taken by community brokers. Across all three themes, stakeholders talked about many factors related to the needs for autonomy, competence and relatedness, all of which underpin the development of autonomous motivation within self-determination theory [14]. Common strategies to promote autonomy satisfaction include; ensuring people perceive a personally meaningful rationale for a given behaviour, that they feel their perspective and potential barriers to change have been acknowledged, promoting choice and agency [29, 30], and that they trust those setting the rules. All four of these strategies were evident in how stakeholders talked about their role. For example, community brokers emphasised how they acted as a trusted source of information (Theme 1), and were able to tailor messages to meet the needs of hyper-local contexts by using their understanding of local community priorities and preferences to adapt the style, language and content of the rationale for adhering to guidance in a way national government could not (Theme 2). Theme 3 illustrated the consideration given by community brokers to preserving people’s choice and agency where they could, talking of their desire for greater delegated decision-making power over the implementation of regulations at the local level, allowing them to arrive at requirements that would be more appropriate (i.e., acknowledging the diversity of needs across their populations), and feasible for both enforcement and adherence. They demonstrated a keen awareness of the importance of keeping the trust of their local communities, and the greater potential for forming trusted relationships locally than nationally.

Insight into how relatedness can be supported was also evident throughout the interviews, underpinned and made possible by community brokers being already deeply embedded within their local community, as trusted and familiar players (often seen as ‘one of us’). Support for autonomy and relatedness can often be intertwined [19, 31] as was indicated by community brokers who felt it was not just the tailored information they provided that was important in promoting adherence, but the fact it came from them, as trusted contacts with whom people felt a connection. Community brokers remarked on the particular importance of fostering a sense of community and of “being in it together” (Themes 2 and 3) when many people were feeling isolated and lacked daily contact with their usual social connections.

Support for the need for competence was also reflected in community brokers’ accounts; while the behaviours themselves that were the subject of health messaging were relatively straightforward (e.g. hand washing, wearing a mask, staying at home), the challenge people experienced was in keeping up to date with changing rules, digital access and literacy, and feeling able to maintain their adherence in the face of others in their social networks not doing so. For example, in Theme 1, community brokers talked about how they tried to aid people’s understanding and subsequently knowledge of how to adhere to guidelines by identifying for them what were the most important aspects, and on occasion, being available to answer queries. In Theme 2, community brokers raised the challenge of digital literacy (the ability to find, evaluate, create and communicate information), and how they provided support to those who were not able to engage using technology. Theme 3 reflected the need for adaptation of guidance and support according to different levels of competence both in relation to adherence to COVID-19 measures, and continuing activities of daily living while still complying with national guidance. Thus, community brokers reflected that their efforts needed to be directed towards supporting people’s abilities in response to these wider issues, involving techniques such as targeted outreach, providing simplified summaries and making themselves available as a single source of authoritative advice.

There is very little previous research conducted into the effect of public health policy on the satisfaction of the needs of autonomy, competence and relatedness. As a result, techniques that could operate at this community level are missing from the taxonomies that are commonly drawn on for intervention design [29, 32]. Our findings provide novel evidence of how the approaches recommended by theory [11, 19, 33] can be put into practice to promote the supportive communication of public health policy. Given the importance within public health of addressing the wider determinants of health, and focusing interventions beyond the individual level to communities, this translation of theory to policy level is particularly important to enable an evidence-based approach.

Looking beyond the health field, past work studying the application of SDT to public responses to pro-social behaviours, based on messaging at a national (or policy) level is primarily reported in relation to pro-environmental behaviours [34–36]. This body of work shows the importance of autonomous motivation in predicting future pro-social behaviours, even in the presence of established current habits [35]. In this setting, by manipulating messaging about the potential outcomes of adopting and maintaining environmental behaviours (which may require effort or sacrifice), framing the aims or goals for a policy in terms of intrinsic compared with extrinsic outcomes (in this case environmental gain, rather than personal financial benefit), leads to stronger autonomous motivation and longer term behaviour change [34, 36]. Our findings extend this work to contribute novelty to this field in two ways; firstly by providing a naturally occurring case study of the importance of who it is that manages the implementation of guidance or regulations (national vs. local and hyper-local levels) and how it is implemented (rigid vs. tailored), can influence our ability to provide need support as part of policy implementation. Secondly, by using a theoretical framework to describe stakeholders’ accounts of the key elements that enabled them to support adherence, we are better able to explore and compare these approaches in other settings and types of behaviour.

Overall, our findings suggest that the benefit of working alongside community organisations as message brokers in implementing public health information during the COVID-19 pandemic could be understood through their ability to provide support for public needs for autonomy, relatedness and competence in a way that more distant national actors cannot. When top-down public health policies are necessary (as was the case in the COVID-19 pandemic), working with community brokers and providing greater decision-making power at a local level could enhance behavioural adherence. As such, future research is warranted to better understand the relationships and methods of communication between national public health, local public health, community organisations (brokers) and local community members, with a view to increasing the reach and efficacy of public health interventions.

## Implications

While our findings are based on data collected during the COVID-19 pandemic, their implications extend beyond emergency response situations. Many longer-term public health challenges such as vaccination uptake, disease prevention, healthy lifestyles and reducing health inequalities depend on sustained behaviour change rather than one-off compliance. Our findings suggest that community brokers play a crucial role in supporting these behaviours through communication environments which nurture psychological needs around relatedness, autonomy and competence framed within SDT.

A key implication is the need to invest in the soft-infrastructure of public health [37], and recognise that community organisations such as schools, VCFSEs and local businesses are important enablers towards improved public health. Many of our participants acted as brokers incidentally due to their trusted role within their community, not as a result of formal public health roles. Consequently, community-brokers cannot be easily substituted by local authority staff who may lack resources, capacity and established relationships based on trust. As such, national and local governments should recognise the contribution of community brokers and strengthen these roles through appropriate funding, training, access to evidence-based resources and ongoing support. Such support should build community capacity and be seen as complimentary to improve public health, not substitutionary. This also aligns with ambitions outlined in the NHS 10 Year Plan (2025) whereby neighbourhood working is central to delivering integrated preventative and person-centred care [3]. It therefore underscores the importance of collaborative working between statutory bodies and trusted community leaders towards health and wellbeing.

Rather than delegating responsibility for public health decision making, our findings suggest that community brokers should be supported to contextualise and communicate public health information within their own communities. This requires timely access to information, regular communication with public health teams, adaptable resources and opportunities to gain clarification when evidence evolves [13]. Furthermore, supporting brokers in this way could also encourage two-way communication enabling local authorities to benefit from public health information cascading downwards, while local insight simultaneously filters upwards, thus informing local public health priorities [13]. This could be invaluable when considering specific communities of interest and recognises that community brokers are not replacements for public health expertise, instead, they are trusted ‘bridges’ who are able to translate and meaningfully engage with their communities.

Finally, the concept of community should be broadened to better reflect hyperlocal community assets. Local businesses, VCFSEs, schools, sport clubs, cafes, shops and community spaces could all be sites for public health related conversations, information exchange and proactive engagement. Appropriate investment into these community assets could strengthen public health initiatives whilst also enhancing the reach and relevance ofcommunications.

## Limitations

While we highlight the important role of community brokers, our findings should be viewed in light of several limitations. Firstly, the study was set in one area of South West England. Confidence in the transferability of findings would be enhanced by further work exploring the ways in which local organisations work in partnership with public health teams. Secondly, while we were able to obtain a broad range of perspectives across different types of community organisations, our sampling technique meant that only organisations known to the local authority were invited to take part which could mean alternative perspectives and experiences were not captured.

## Conclusion

This study demonstrates the importance of community brokers during the COVID-19 pandemic. Brokers were embedded within their respective communities and bridged the gap between national COVID-19 guidance and legislation and localised implementation. Their effectiveness can be viewed through the lens of self-determination theory, particularly in how their position within the community allowed them to support individuals’ needs for autonomy, relatedness and competence in fostering more autonomous and long-lasting motivation towards transmission control behaviours. The strategies they reported using to achieve this included the development of trusted status, fostering a sense of community and shared benefit from adherence, tailoring and simplifying of messages, and proactive outreach. Acknowledging and strengthening the role of community brokers in future public health challenges may encourage more responsive, accessible and meaningful health communication strategies and enable more sustained adherence to these healthy messages and behaviours.

## Supplementary Information

Supplementary Material 1.

## Data Availability

Anonymised data used and/or analysed during the current study are available from the corresponding author on reasonable request.

## Footnotes

1 Dominic Cummings was the UK prime minister’s most senior adviser at this time, and drew public criticism for driving 260 miles from London to North East England to stay with family after his wife had become unwell. The British public had been expressly instructed by the Government to self-isolate for two weeks after experiencing coronavirus symptoms. Many avoided travel even at the cost of missing important events such as the funerals of loved ones [20].

## Abbreviations

CHW: Community-health workers
SDT: Self-Determination Theory
VCFSE: Voluntary, community, faith and social enterprise
