# Supplementary material for: Exploring the role of community-based knowledge brokers in public health communication through the lens of self-determination theory: a qualitative study
Source: BMC Public Health. 2026 Aug 6;26:3174. doi: 10.1186/s12889-026-28665-7 (PMC13729310; doi:10.1186/s12889-026-28665-7)
Supplement: Supplementary file 1 [file 12889_2026_28665_MOESM1_ESM.docx]

*Introduction*

Introductions

Hello, We are doing a study with [Local Authority] to help improve our understanding of how to stay safe during the COVID-19 pandemic. We are conducting an evaluation about COVID-19 and adhering to government guidelines, specifically those relating to social distancing, testing and self-isolation.

The purpose of this part of the project is to talk to those who work with or volunteer with partner organisations who work primarily in [location], to understand the barriers people may face in following the rules on social distancing, testing, and self-isolation and to try to identify the best ways to enable and encourage people to do this.

*Interview questions*

1. Can you give me a brief overview of the work your organisation does with local people?
2. Thank you – can we talk a bit now about the current COVID-19 pandemic, and the main challenges those that use your services have faced over this time. What sorts of experiences have these people had? First can we talk about how you think they have coped with **social distancing**,
   a. Are there any particular examples or stories that stick in your mind about this? Perhaps things you have come across yourself or that you have heard from other people - what stands out to you? *[probe for detail – What happened? Who said what? When was this? Were they a part of this story from the beginning? How did it start? Was there anything unusual about these interactions? Was this a common issue? What have they heard from colleagues? What do they think about the experience? Allow for multiple stories*. After social distancing cover:
   b. Now can we talk about **self- isolation**, (*when you do not leave your home because you have or might have coronavirus?*)
   c. Finally, can we talk about **testing** (*in order to find whether someone has covid-19?)*
3. What have been your observations of how well people have followed the government guidelines of **social distancing**? [Then **self-isolating** and **testing**? [*probe again for stories of specific experiences – what do they think about it? Why do they think this is the case?*]
4. Over the course of the pandemic have you seen any changes at all in how people are following the guidelines? If yes, what has changed? Why do you think this is?
5. What do you think **hinders** people’s ability to adhere to the guidelines? [*expect discussion of structural and social barriers as well as communication barriers. Do they have an opinion on how to remove the barriers they’ve identified?*]
6. What do you think **helps** people’s ability to adhere to the guidelines? [*expect discussion of structural and social barriers as well as communication barriers. Do they have an opinion on how to build upon the helpful factors?*]
7. Is there anything you would like to add that you think we might have missed about how the people you come into contact with are managing guidelines around **social distancing (self-isolation/testing)**
